# Psychological Signs as the Only Presentation of Wilson’s Disease in an 11-Year-Old Boy 

**Published:** 2018

**Authors:** Mehran BEIRAGHI TOOSI, Javad AKHONDIAN, Farah ASHRAF ZADEH, Nahid DONYADIDEH, Asma JAVID

**Affiliations:** 1 Department of Pediatric Neurology, Ghaem Hospital, Mashhad University of Medical Sciences, Mashhad, Iran

**Keywords:** Wilson disease, Psychological symptoms, Childhood, Iran

## Abstract

Wilson’s disease (WD) is a rare autosomal recessive disease due to copper metabolism disturbance. The clinical presentation spectrum of Wilson’s disease is wide and initial findings of the disease depend on the organ involved. Neurologic disorders can develop insidiously or precipitously with intention tremor, dysarthria, rigid dystonia, Parkinsonism, deterioration in school performance or behavioral changes. This article is presenting an 11-yr old boy with chief complaint of falling and upper limb spasm. He referred to the Neurology Department, Ghaem Hospital, Mashhad, northeastern Iran in 2016. His symptoms began from 6 months earlier as mood instability (prolonged spontaneous crying). He was also suffering from occasionally tremor and micrographia. Initial investigations were normal and with diagnosis of depression and psychiatric problems, he had undergone treatment with fluoxetine and risperidone. Wilson’s disease should be considered in the diagnosis of all children with psychiatric and musculoskeletal symptoms.

## Introduction

Wilson’s disease (WD) (hepatolenticular degeneration) is a rare autosomal recessive disease due to copper metabolism disturbance (1). Reduced excretion of copper in bile leads to accumulation of excess copper and degenerative changes mainly in the liver and brain (2). WD affects between 1:30000 and 1:50000 individuals. It is progressive and potentially fatal disease (1).

The clinical presentation spectrum of WD is wide and initial findings of the disease depend on the organ involved. Hepatic involvement findings are observed more commonly and usually appear during first three decades of life (1, 3). The approximate incidence of neurological symptoms in children is 15% and predominant after 20 yr of age (1, 3). Most patients with first presentation of neurological or psychiatric signs are older than those with hepatic features alone. Most of them have asymptomatic liver disease at the time of CNS presentations (4).

Neurologic disorders can develop insidiously or precipitously with intention tremor, dysarthria, rigid dystonia, Parkinsonism, choreiform movements, lack of motor coordination, deterioration in school performance or behavioral changes (1). Psychiatric manifestations include depression, personality changes, anxiety or psychosis (1). Serum ceruloplasmin and 24-h urinary copper excretion check out are useful tools to identify most pediatric patients with WD (5). Serum ceruloplasmin <20 mg/dL and 24-h urinary copper >40μg/ 24h are recommended values suggestive of WD for pediatric screening (6).

Here we present a cases of an 11-yr old boy with chief complaint of falling and upper limb spasm.

## Case presentation

In Mar 2016, an 11-yr-old boy with chief complaint of falling and dystonia of upper limbs referred to the Neurology Department of Ghaem Hospital, Mashhad, northeastern. His symptoms began from 6 months earlier as mood instability (prolonged crying without any reason). He was also suffering from occasionally tremor and micrographia. Initial workups were normal and with diagnosis of depression and psychiatric problems, he had undergone treatment with fluoxetine and risperidone. After 6 wk his symptoms got worse so olanzapine and lamotrigine were added to his treatment. Previous symptoms were progressed besides abdominal pain and involuntary irregular movements of extremities showed up. 

Informed consent was taken from the parents and the study was approved by Ethics Committee of the hospital.

The past medical history was unremarkable. His parents were cousin and he had two younger siblings. His grandmother died in the third decade of her life with neurologic symptoms but unknown etiology. 

Complete physical examination performed and revealed motism, depression, fluctuation in his mood, athetosis, tremor, drolling, spasticity in limbs, exaggerated deep tendon reflexes and downward plantar reflexes. 

**Fig. 1:**
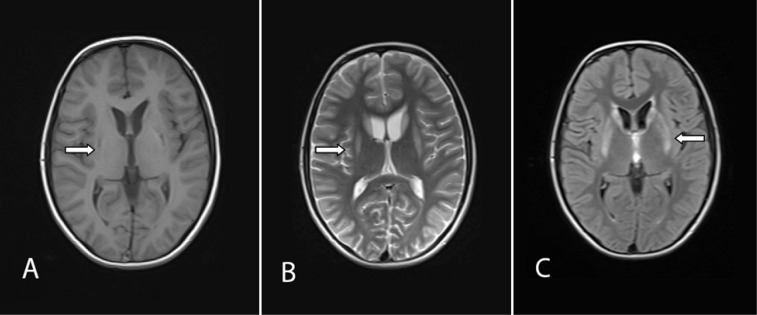
Brain Magnetic Resonance Imaging; Axial views. (A) T1- weighted sequence: decreasing signal intensity of bilateral putamen. (B) Hyper signal lesions of bilateral caudate and putamen nucleuses in T2 - weighted sequence. (C) Fluid Attenuated Inversion Recovery (flair) sequence. Bilateral involvement of putamen and head of caudate nucleus is seen

For more consideration, Brain Magnetic Resonance Imaging (MRI) was done. Increased signal intensity in bilateral caudate and putamen nucleuses in T_2_ – weighted images and decreasing signal intensity without restriction in T_1 _– weighted images were reported (Figure 1). Electroencephalography (EEG) had normal pattern (Table 1).

A Kayser-Fleischer (KF) ring was reported in ophthalmologic examination. The diagnosis of WD confirmed and therapy with high dose of zinc sulfate initiated. The patient’s siblings were screened for WD and their serum ceruloplasmin were in normal range (47 mg/dl and 43 mg/dl).

## Discussion

We described an 11-yr-old boy with WD who initially presented with mood disorders and muscle spasms who was under the psychiatric treatments and showed the importance of ruling out medical causes of psychiatric signs before making a diagnosis. Psychiatric manifestations were reported in 30%–50% of the patients prior to a diagnosis of WD. “Irritability, Incongruous behavior, depression, and cognitive impairment were the most prevalent psychiatric symptoms among these patients” (7, 8).

**Table 1:** The laboratory data for Wilson's disease child

**Characteristics**	**Value**	**Characteristics**	**Value**
Aspartate aminotransferase	45 U/L (5–40 U/L)	24 hour urinary copper	95 micg/24h (15-70 micg/24h)
Alanine aminotransferase	38 U/L (5–40 U/L)	Serum lactic acid	10.2 mg/dl ( 4.5-20 mg/dl)
Serum ceruloplasmin	17.6 mg/dl (15-60 mg/dl)	Serum pyruvate	8 mg/dl (.3-1 mg/dl )
Serum ammonia	49.1 mg/dl (4-80 mg/dl)	Cerebrospinal fluid	Normal

Cognitive impairment can be subtle and only identified by the family after the WD diagnosis (9). It is categorized into two main groups: a frontal lobe syndrome and subcortical dementia (10).

Manifestations of frontal syndrome are emotional lability, impulsivity, promiscuity, apathy, impaired social judgment, decreased attention and pseudobulbar features. Slowness of thinking, memory loss and executive dysfunction are some characterizations of subcortical dementia (10, 11). The most common psychiatric symptoms reported at the time of diagnosis are personality changes, irritability, incongruous behavior and depression (7). Chief complaint of muscle spasm is not included as presenting symptoms of WD in textbooks or national guidelines, which can misguide initial evaluation (12, 13).

In a study, 12 children were evaluated with a diagnosis of WD and central nervous system involvement. The mean time between the diagnosis of WD and occurrence of neurological findings was 42.2±30.6 months. About 25% patients presented with neurological symptoms and diagnostic investigations revealed WD diagnosis (14).

**In conclusion, **although WD in almost all children presents as hepatic dysfunction, it should also be considered in the diagnosis of all children with psychiatric and musculoskeletal symptoms. Consequently, before the diagnosis of psychiatric disorders according to DSM-5, physicians should rule out organic disorders especially WD (15); determination of serum ceruloplasmin levels and 24-h urinary copper excretion is an effective and noninvasive screening way.
